# Preparation of diglycolamide polymer modified silica and its application as adsorbent for rare earth ions

**DOI:** 10.1080/15685551.2018.1564425

**Published:** 2019-01-05

**Authors:** Zhe Liu, Yu Liu, Aijun Gong

**Affiliations:** a School of Chemistry and Biological Engineering, University of Science and Technology Beijing, Beijing, China; b Beijing Key Laboratory for Science and Application of Functional Molecular and Crystalline Materials, University of Science and Technology Beijing, Beijing, China; c Institute of Biotechnology, Daqing Branch of Heilongjiang Academy of Science, Daqing, China

**Keywords:** Rare earth, adsorption, polymer-grafted, diglycolamide

## Abstract

Three novel diglycolamide monomers were synthesized and polymerized on silica. The diglycolamide polymer grafted silica were used as adsorbents for rare earth ions. The effects of acid concentration, structure of monomer, initial solution concentration, contact time and coexisting ions on adsorption of rare earth ions were investigated in detail. It was shown that the adsorption capacity increased with increasing acid concentration. Three adsorbents exhibited selectivity for middle and heavy rare earth over light rare earth in different extent. The adsorbent prepared from the monomer having the largest alkyl substituent showed the lowest adsorption capacity but the highest selectivity for different rare earth elements (REEs). Adsorption data were well fitted to the Langmuir isotherm and pseudo-second-order models. The presence of high concentrations (100 fold) of coexisting metal ions, K(I), Cr(II), Cu(II) or Fe(III), does not decrease the adsorption for rare earth ions seriously.

## 1. Introduction

Rare earth elements play an important role in modern technology. However, during the exploitation and usage process, more and more REEs are getting into the environment, such as soil and water [1–3]. The determination of trace REEs in biological and environmental samples has great environmental and biological significance. However, in some cases, the low level of REEs in samples is not compatible with detection limits of the commonly used analytical instrument, ICP-AES. And major constituents such as organic compounds and inorganic salts usually cause matrix effects, making the matters worse [4–6]. To overcome these problems, preconcentration and separation process are prerequisite. Among the various technologies, such as solvent extraction, precipitation and ion-exchange, adsorption has the advantage of ease of operation, lower consumption of hazardous reagent and reusability.

Various adsorbent such as polymer materials [7,8], carbon materials [9,10], and silica materials [11,12] have been studied. To selectively adsorb REEs, small molecules bearing chelating groups such as β-diketone [13], EDTA [14], are often used to modify the material surface. Diglycolamide (DGA), containing ether linkages between two amide groups, display high affinity for REEs and have been used as extractant [15,16]. Inspired by these work, many research had been focused on DGA modified materials for the selective adsorption of REEs [17–20]. However, adsorption capacity is not very satisfactory due to only one chelating functionality per anchoring molecule is incorporated at the surface, providing limited surface functional group density. It is well known that the adsorption capacity increases with ligand loading on the support, which is limited by the number of functional groups on the support surface (e.g. silanol group for silica gel). The specific surface area increases with decreasing average pore size. However, decreasing the pore size will reduce adsorption rate, hence increasing both adsorption capacity and adsorption rate simultaneously is difficult.

Surface initiated radical polymerization (SIRP) provides a convenient method for increasing the surface functional group density by forming polymer brushes on solid surface. Although polymer brushes modified materials have been extensively studied for adsorption of transient metals or heavy metals, REEs has rarely been concerned with [21–24]. In this paper, we designed and synthesized three vinyl monomers bearing the DGA group. Polymer brushes were grafted on the silica surface via SIRP. The modified silica was used to efficiently absorb REEs from aqueous solution. Batch sorption experiments were conducted to investigate their sorption behaviors.

## 2. Experiment

### 2.1. Preparation of polymer grafted silica

**Figure 1.**
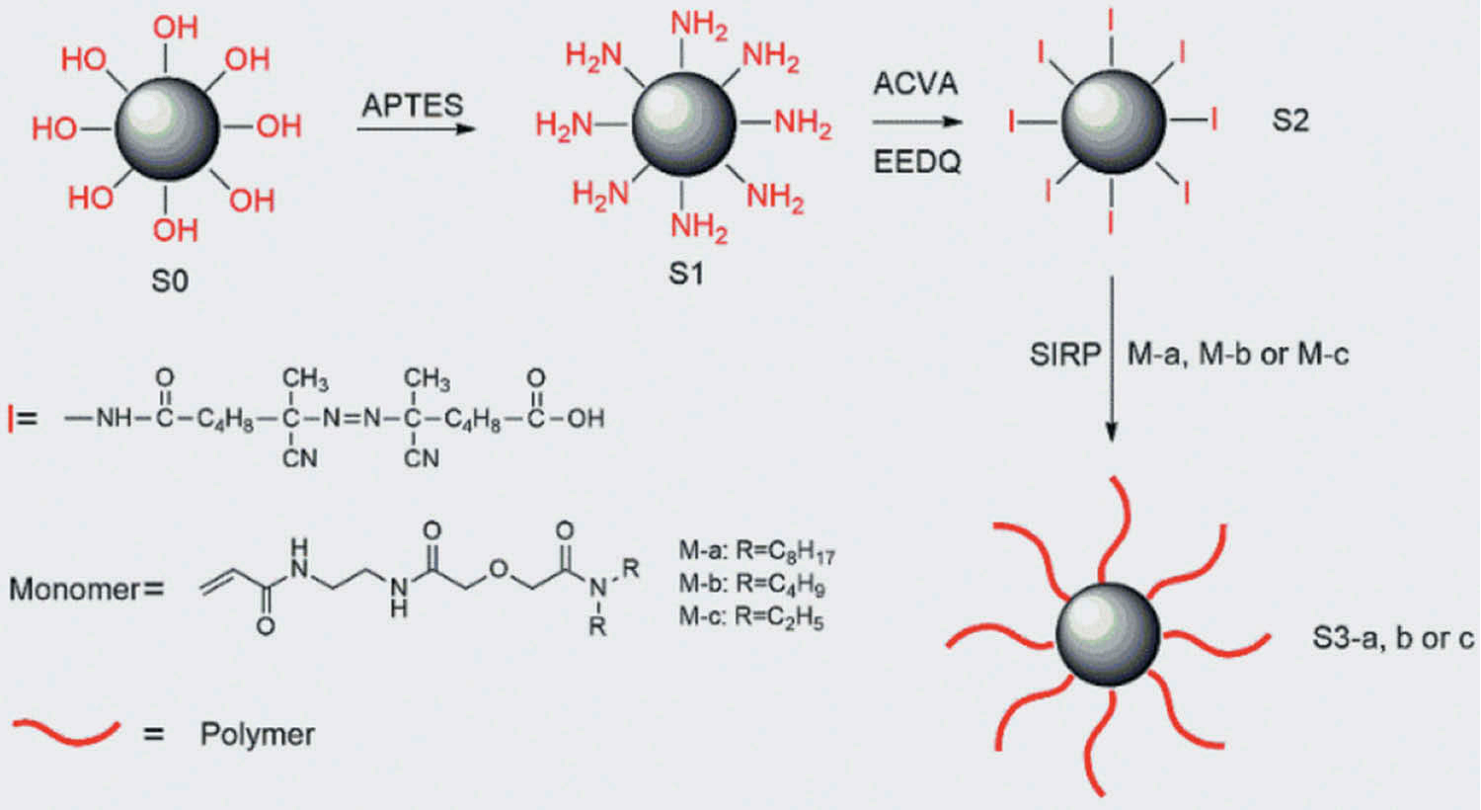
SiO_2_ modification and SIRP.

The route of SIRP was given in Figure 1 and the detail was as follows, in which the monomers, M-a, M-b and M-c, were synthesized as described in supporting information (SI).

#### 2.1.1. Modification of silica nanoparticles

APTES was immobilized on silica via a silane coupling reaction. Briefly, silica (10 g) and toluene (500 mL) were placed in a flat bottom flask with a reflux condenser and a magnetic stirrer. APTES (10 g) was added to the flask, and the mixture was heated under reflux for 12 h with stirring. After cooling down, the solid product was filtered, washed with toluene and methanol and dried at 40°C in vacuum overnight. The product was denoted as S1.

The ACVA-attached silica was synthesized as follows. ACVA (15 g) and EEDQ (15 g) were dissolved in DMF (100 mL) in a three-necked flask. The solution was bubbled with N_2_ gas for 30 min. S1 (4 g) were then immersed in the solution, and the mixture degassed again for 30 min before commencement of the reaction, which was carried out at 25°C for 30 h under an N_2_ atmosphere. The ACVA-attached silica was washed consecutively with DMF and methanol and dried in vacuo at 40°C for 1 day. The product was denoted as S2.

#### 2.1.2. Polymer grafted on SiO_2_

S2 (1 g), monomer (2 g) and DMSO (5 mL) were added to a Schlenk flask. The mixture was heated at 80°C under N_2_ atmosphere for 10 hours. After the reaction the polymer-grafted silica was washed three times with DMSO, ethanol and acetone to remove any soluble polymer and monomers. The product was then dried in vacuo at 40°C for 1day.

### 2.2. Adsorption experiments

The adsorption experiments were performed by contacting 10 mg adsorbent with 10 mL of REE-containing aqueous solution for certain time. The concentration of rare earth element in the residue solution was determined by ICP-OES. The adsorption capacities (mmol g^−1^) were calculated as

(1) $$q=\frac{C_{0}-C_{e}}{M}V$$

where *C*_0_ and *C*_e_ are the initial and equilibrium concentrations (mmol L^−1^) of REEs, respectively, while *M* and *V* represent the weight of the adsorbent and volume of solution, respectively. All the tests were conducted in duplicate, and only the average values were given

## 3. Results and discussion

### 3.1. Characterization of adsorbents

**Figure 2.**
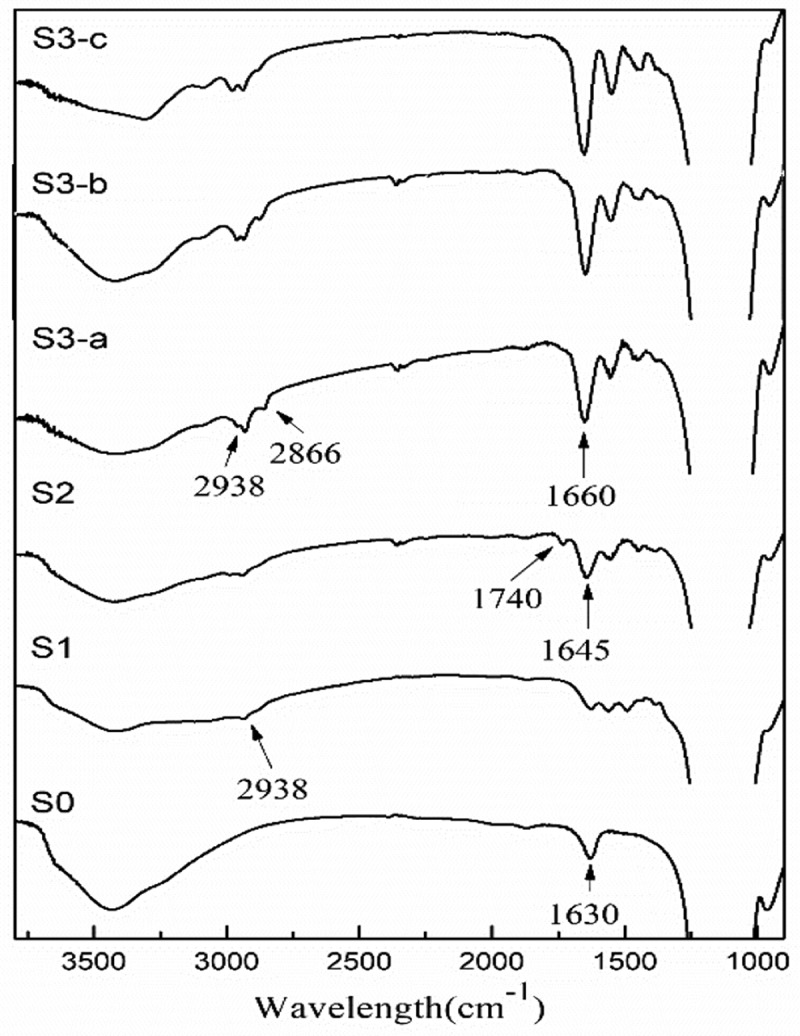
Infrared spectra (left) and XPS wide-scan spectra (right) of pure SiO_2_(S0), APTES-SiO_2_(S1), ACVA-SiO_2_(S2) and DGA polymer-grafted SiO_2_(S3).

Figure 2 presents the IR spectra of S0, S1, S2 and S3. In S0, peak at 1630 cm^−1^ comes from SiO-H bending vibration; In S1, peak at 2938 cm^−1^ is attributed to the C***–***H stretching of CH_2_ and CH_3_, demonstrating that APTES is successfully immobilized on the surface of silica nanoparticles. In S2, peaks at 1740 and 1645 cm^−1^ are ascribed to the vibration of C=O in carboxyl and amide group, respectively, confirming that initiator was attached successfully. In S3-a, S3-b and S3-c, each shows a strong peak at 1660 cm^−1^ and medium peaks at 2938**–**2866 cm^−1^, which can be ascribed to the amide and CH_2_ group from the monomers, demonstrating that polymerization was initiated on the silica.

**Figure 3.**
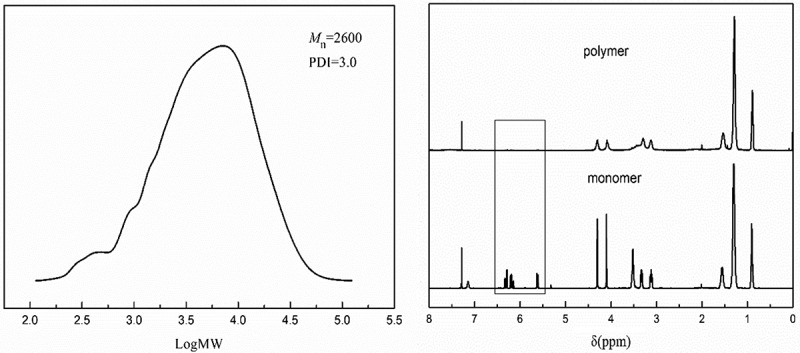
GPC curves (left) and ^1^H NMR spectrum (right) of the polymer cleaved from S3-a.

To further confirm the success of SIRP, S3 was treated with HF as in literature [25], and the product cleaved from the silica was characterized by GPC and ^1^H NMR as shown in Figure 3. Take S3-a as an example, the GPC curves showed that number-average molecular weight(*M*_n_) of the product was 2600. ^1^H NMR showed that the double bond disappeared in the product compared with monomer. These also confirmed that radical polymerization was conducted on the silica.

### 3.2. Acid concentration effect on adsorption

**Figure 4.**
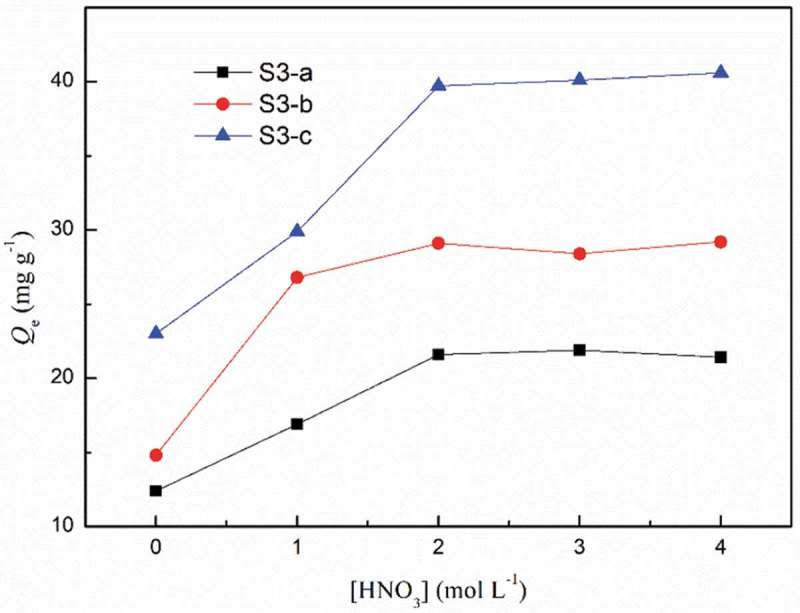
Effect of acid concentration on the adsorption of Eu(III). (*C*_0_ = 50 mg L^−1^, *V* = 10 mL, *W* = 10 mg, time = 6 h).

The Acid concentration has great influenced on adsorption. In this experiment, Eu were selected as representative to explore acid concentration effect. As shown in Figure 4, the adsorption capacity increased with the increase of acid concentration and reached a plateau when HNO_3_ concentration was raised. Similar results can also be found in previous works relating DGA modified materials [26,27]. This is probably because DGA is a neutral ligand and binds neutral species, NO^3−^ is indispensable to form the stable neutral complex as shown [15]:

$$M^{3+}+nL+\;3\;NO^{3-}\;↔M(L{)}_{n}(NO_{3}{)}_{3}$$

where M is metal ion, L is ligand. So, increasing NO^3−^ concentration shifts the equilibrium to the right.

In the following experiments, the acid concentration was kept at 2 mol L^−1^. To be noticed, the adsorption capacity for three adsorbent were S3-a<S3-b<S3-c. Adsorption capacity was related to the amount of binding site, i.e., the DGA group. TG curves (See Figure S8 in SI) revealed that the polymer content of S3-c was the most and so was the amount of DGA groups.

### 3.3. Adsorption kinetics

**Figure 5.**
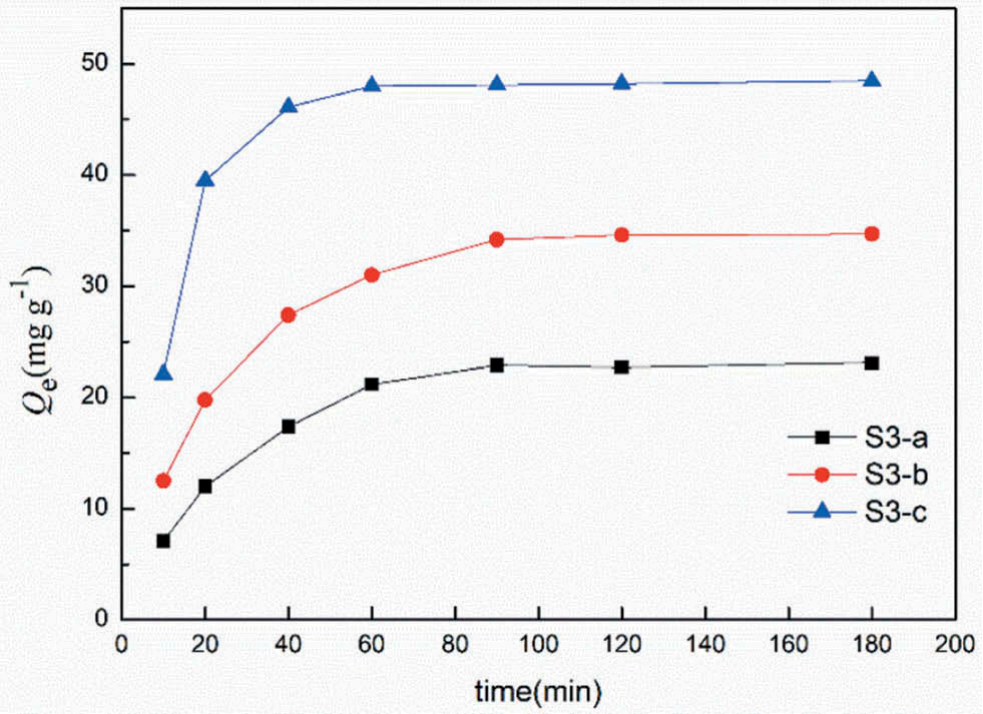
Effect of contact time on the adsorption capacity for Eu(III). (*C*_0_ = 150 mg L^−1^, *V* = 10 mL, *W* = 10 mg, [HNO_3_] = 10^−2^ mol L^−1^).

The effect of the contact time on Eu(III) adsorption with the synthesized adsorbents from solution is presented in Figure 5. The adsorption amount of Eu(III) increased with increasing contact time. The adsorption was very quick at the beginning then proceeded slowly until equilibrium in 180 min.

The adsorption kinetics was analyzed to elucidate the possible rate-determining step of the adsorption process, and two typical kinetic models, pseudo-first order (Equation (2)) and pseudo-second order (Equation (3)) models have been employed to fit the experimental adsorption data.

(2) $$ln\;(Q_{e}-Q_{t})=ln\;Q_{e}-k_{f}t$$

(3) $$\frac{t}{Q_{t}}=\frac{t}{Q_{e}}+\frac{1}{k_{s}Q_{e}^{2}}$$

Where *k_f_* is the pseudo-first order rate constant, *k_s_* is the pseudo-second order rate constant. The correlation coefficient kinetic parameters are listed in Table 1. The second-order kinetic equation gives the best values for R^2^. In addition, the experimental *Q*_e_ values agree well with the calculated *Q*_e,cal_ for the pseudo-second order kinetic model,

**Table 1.** Modeling of adsorption kinetics using pseudo-first and second order models.

	Pseudo-first order			Pseudo-second order		
Sample	*k*_f_ (min^−1^)	*Q*_e,cal_ (mg g^−1^)	R^2^	*k*_s_ (g mg^−1^ min^−1^)	*Q*_e,cal_ (mg g^−1^)	R^2^
S3-a	0.024	18.9	0.984	0.038	26.5	0.997
S3-b	0.040	38.6	0.969	0.026	39.2	0.996
S3-c	0.043	24.9	0.821	0.019	52.1	0.994

**Table 2.** Modeling of adsorption isotherms using the Langmuir and Freundlich equations.

	Langmuir			Freundlich		
Adsorbent	*Q*_max_ (mg g^−1^)	b (L mol^−1^)	R^2^	*K*	n	R^2^
S3-a	23.9	0.183	0.987	6.403	3.175	0.822
S3-b	35.0	0.200	0.996	8.825	2.921	0.832
S3-c	59.4	0.221	0.985	13.428	2.442	0.916

**Table 3.** Separation coefficients between light, medium and heavy REEs.

sorbent	*K*_La_	*K*_Tb_	*K*_Lu_	*D*_Tb/La_	*D*_Lu/La_	*D*_Tb/Lu_
S3-a	0.008	0.238	0.128	29.465	15.850	1.859
S3-b	0.080	0.360	0.302	4.504	3.780	1.191
S3-c	0.234	0.485	0.515	2.068	2.196	0.941

so the pseudo-second order kinetic model fits the experimental results more accurately than the pseudo-first-order kinetic model for all materials. This indicates that the surface chemical sorption could be the rate-determining step.

### 3.4. Adsorption isotherms

**Figure 6.**
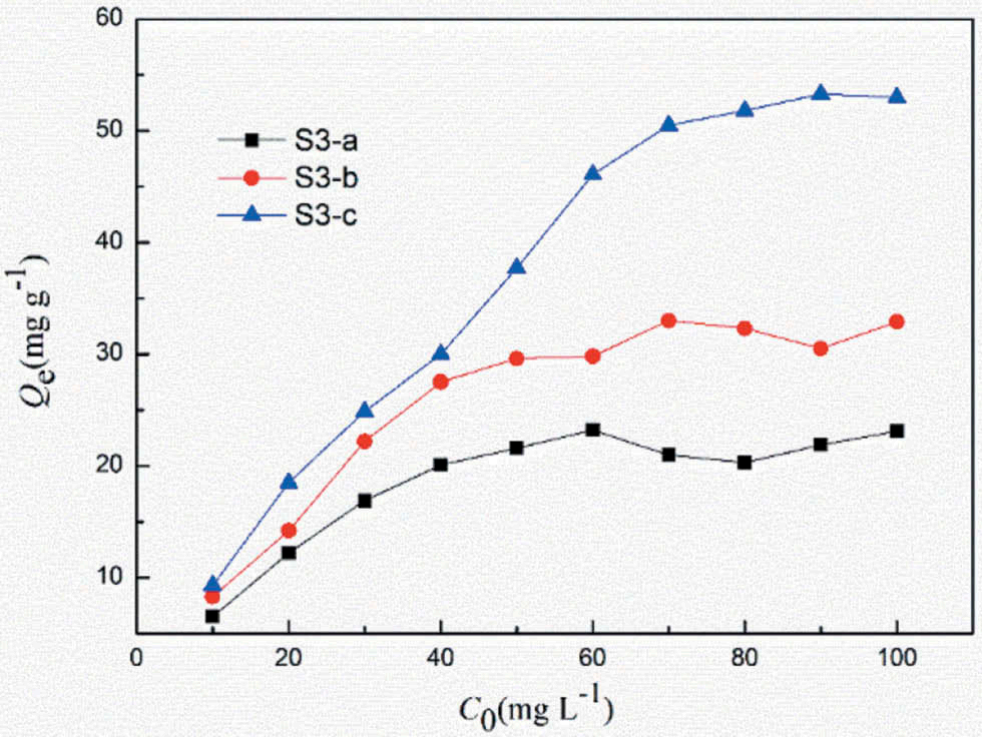
Effect of initial metal ion concentration on the adsorption capacity for Eu(III). (*V* = 10 mL, *W* = 10 mg, [HNO_3_] = 10^−2^ mol L^−1^, time = 6 h).

Adsorption under different initial REE concentration were conducted. As shown in Figure 6, for all materials, the adsorption capacity increased remarkably with increasing *C*_0_ then tended to level off at certain points. Equilibrium relationships between the adsorbent and adsorbate are usually described by the Freundlich and Langmuir isotherms which define the ratio between the quantity adsorbed and that remaining in solution at a fixed temperature at equilibrium and show the sorption capacity of the adsorbent. The widely used Langmuir and Freundlich isotherm have found successful application in many adsorption processes and assumes complete monolayer coverage bound on the surface at high equilibrium metal ion concentration. It is expressed as

(4) $$\frac{C_{e}}{Q_{e}}=\frac{C_{e}}{Q_{max}}+\frac{1}{bQ_{max}}$$

(5) $$log\;Q_{e}=log\;K+\frac{1}{n}log\;C_{e}$$

Where *Q*_m_ (mg g^−1^) is the maximum amount of metal ion adsorbed from solution concentration, b (L mol^−1^) is Langmuir constant related to the binding sites affinity, *Q*_e_ (mg g^−1^) is equilibrium adsorption capacity. *K* (mg g^−1^) and n are Freundlich constants, representing adsorption capacity and adsorption intensity of the system

According to the values of the relative coefficients as shown in Table 2, Langmuir adsorption isotherm fit with the experimental data better. The Langmuir sorption isotherm assumes monolayer adsorption, and the adsorption sites are also assumed to be energetically equivalent and distant from each other so that there is no interaction between molecules adsorbed to adjacent sites. It also assumes that all of the binding sites on the sorbent are free sites to be ready for accepting the sorbate from solution.

### 3.5. Adsorption for various REEs

**Figure 7.**
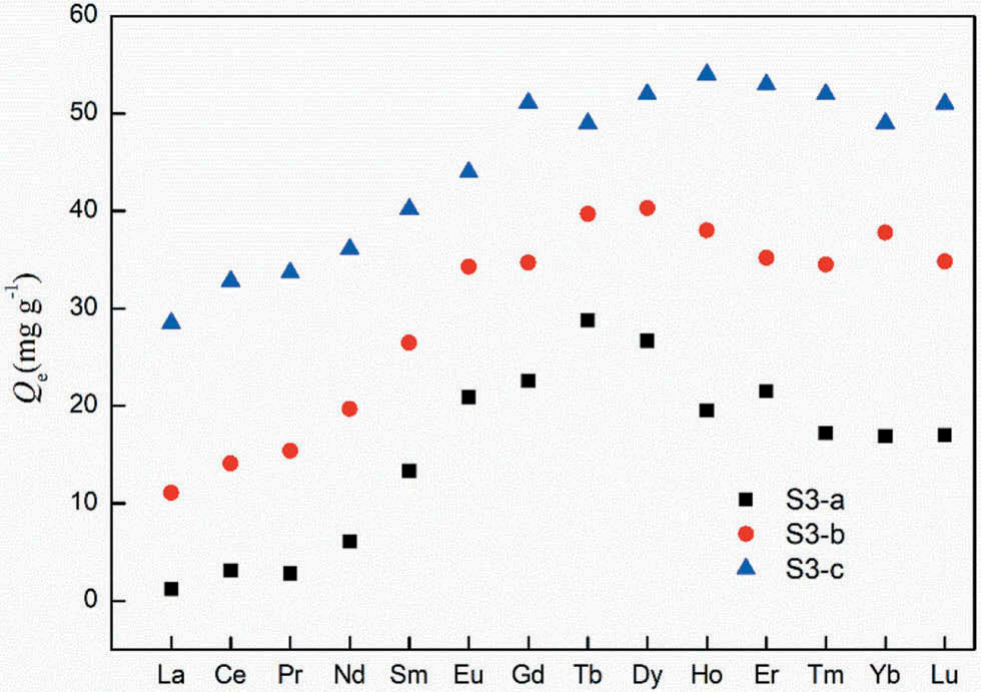
Amount of lanthanoid ion adsorption onto the polymer-grafted SiO_2._ (*C*_0_ = 150 mg L^−1^, *V* = 10 mL, *W*_0_ = 10 mg, [HNO_3_] = 0.01 M, time = 6 h).

The adsorption for all lanthanoid ions (except for Pm) was examined under the condition of *C*_0_ = 150 mg L^−1^. As can be seen in Figure 7, the adsorption capacity of S3-c for REEs can reached over 50 mg g^−1^, which is comparable to that of the materials reported in other literatures [28–32]. S3-a, S3-b and S3-c all prefer to adsorb middle and heavy REEs over light REEs, but in different extent. The affinity of the adsorbent for a specific rare earth ion can be represented by the distribution coefficient *K*_d_ (mL/g), expressed as:

(6) $$K_{d}=\frac{C_{0}-C_{e}}{C_{e}}\times \frac{V}{M}$$

The separation coefficient *D*_1/2_ between metal ions 1 and 2 can be used to describe the selectivity of adsorbent and was calculated as follows:

(7) $$D_{1/2}\;=\frac{K_{d1}}{K_{d2}}$$

La, Tb and Lu were selected as representatives of light, medium and heavy rare earth elements, respectively, and the *D*_1/2_ were listed in Table 3. All *D*_1/2_ of S3-a were obviously large than that of S3-b and S3-c, especially for *D*_Tb/La_ and *D*_Lu/La_. Although S3-a has the lowest adsorption capacity, it shows the best selectivity among different REEs. The difference of *D*_1/2_ may be attributed to the structure of monomers. In M-a, the substituent near the DGA group were two octyl groups, posing the greatest steric hindrance, therefore S3-a show strong preference for the smaller (medium and heavy) REEs. In M-c, the two ethyl groups lead to the least steric hindrance, so S3-c exhibited weaker preference for smaller REEs.

### 3.6. Coexisting ion effect on adsorption

**Figure 8.**
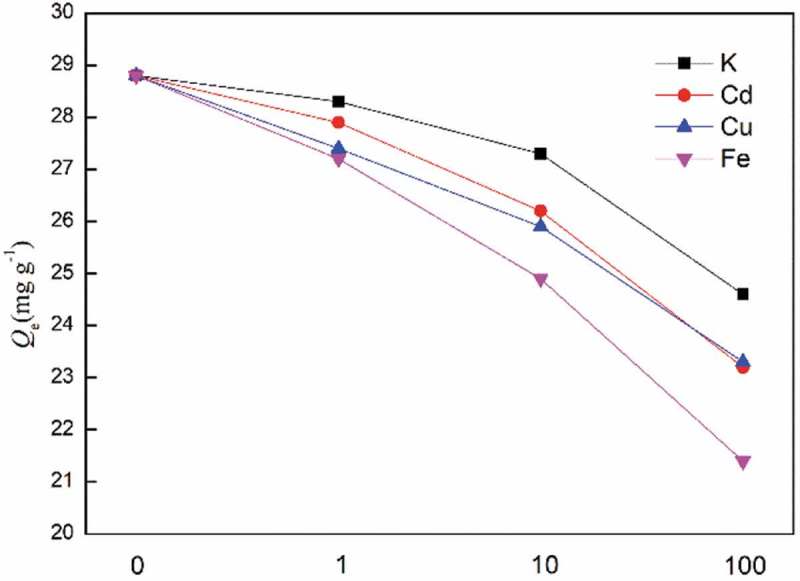
Coexisting ion effect on adsorption. (*C*_0_ = 150 mg L^−1^, *V* = 10 mL, *W*_0_ = 10 mg, [HNO_3_] = 0.01 M, time = 6 h).

High concentration coexisting ions are ubiquitous in real sample and often interfere REEs adsorption, so REEs adsorption by S3-a as an example in the presence of coexisting ions was investigated by adding large amount of KNO_3_, Cd(NO_3_)_2_, Cu(NO_3_)_2_ or Fe(NO_3_)_3_. As can be seen from Figure 8, equal amount coexisting ion has little effect on adsorption capacity and 10-fold coexisting ion decreased adsorption capacity less than 14%. Even 100-fold coexisting ion, except for Fe^3+^, only leaded to less than 20% reduction. Some materials, such as graphene oxide [10], polyamide [33] and poly(acrylic acid) [34], exhibit higher adsorption capacity for REEs, but they adsorb coexisting ions even stronger [35–38]. In this work, the diglycolamide polymer modified silica have excellent anti-interference ability as well as high adsorption capacity.

## 4. Conclusion

In this work, three novel vinyl monomers bearing the DGA group were designed and synthesized. Diglycolamide polymer grafted silica were prepared by SIRP and used as adsorbents for rare earth ions. The effects of acid concentration, structure of monomer, initial solution concentration, contact time and coexisting ions on adsorption of REEs have been investigated. The adsorbents exhibited desirable adsorption capacity, selectivity and anti-interference ability for REEs, suggesting prospects of analytical and industrial applications.
