# Supplementary material for: Preparation of diglycolamide polymer modified silica and its application as adsorbent for rare earth ions
Source: Des Monomers Polym. 2019 Jan 5;22(1):1–7. doi: 10.1080/15685551.2018.1564425 (PMC6327932; doi:10.1080/15685551.2018.1564425)
Supplement: Supplemental Material [file TDMP_A_1564425_SM4854.docx]

**Supporting Information**

**[Preparation of Diglycolamide Polymer Modified Silica and Its Application as Adsorbent for Rare](http://pubs.acs.org/doi/abs/10.1021/cm030334y) Earth Ions**

Zhe Liu^1,2^, Yu Liu^1,2,3^, Aijun Gong*^1,2^

^1^School of Chemistry and Biological Engineering, University of Science and Technology Beijing, Beijing 100083, China

*^2^Beijing Key Laboratory for Science and Application of Functional Molecular and Crystalline Materials, University of Science and Technology Beijing, Beijing 100083, China*

^3^Institute of Biotechnology, Daqing Branch of Heilongjiang Academy of Science, Daqing 163319, China

E-mail: gongaijun5661@ustb.edu.cn

**1 Materials and instruments**

Dicyclohexylcarbodiimide (DCC, 99%), 1-hydroxybenzotriazole (HOBT, 99%), N-boc-ethylenediamine (Boc-EDA, 95%), acryloyl chloride (99%), di-n-octylamine amine (95%), di-n-butylamine (95%), diethylamine (99%), 3-aminopropyl)triethoxysilane (APTES, 99%), 4,4'-azobis(4-cyanovalericacid) (ACVA, 98%), [N-ethoxycarbonyl-2-ethoxy-1,2-dihydroquinoline](http://www.baidu.com/link?url=bIq5gwmgGWp2FmTaCWj_1rl9lESfcXGPAtSyo_BRr8oXz1ZcwQqwdazMWvKCewm6i3ZZb81YyQ1zLJO678iPntXGAiHni3OVTLmTKnP1LFS_z8KvsQFHfNLqnegOvV2Q) (EEDQ, 98%) and SiO_2_ (50 ± 5 nm, 185 ± 30 m^2^ g^-1^) were all used as received.

Fourier transform infrared spectroscopy was performed on Thermo Scientific Nicolet iS10. NMR spectra were recorded with a Bruker DMX-400 MHz. Ions concentration was determined by [Agilent 5100 ICP-OES](https://www.baidu.com/link?url=_lezZJVsdv_N4fgKyiUfD0KoR9DpkCRdIz2QLqX2MuqLDAg2E7Bb3R-ldrjyrB2ts8N3OXQATSDqCqeztmm1jdcT-dxqQQY6Z7UKwtEtQe3&wd=&eqid=de38e97b0003daba000000025c18b706). Polymer molecular weight was determined by Waters 1515 equipped with two PLgel 5 μm MIXED-C columns using THF as the eluent.

**2 Synthesis of monomer**





**Figure S1.** Synthesis route of monomer

2.1 Synthesis of a1

Diglycolic anhydride (30 g) was dispersed in CH_2_Cl_2_ (300 mL) in an ice-bath, di-n-octylamine (62 g) dissolved in CH_2_Cl_2_ (50 mL) was dropped to the mixture and stirred at room temperature overnight. The solution was poured into dilute hydrochloric acid and the mixture was subjected to liquid separation. The aqueous phase was extracted CH_2_Cl_2_ three times, the organic phases were combined, concentrated and recrystallized to give a1 as white crystal (70% yield).


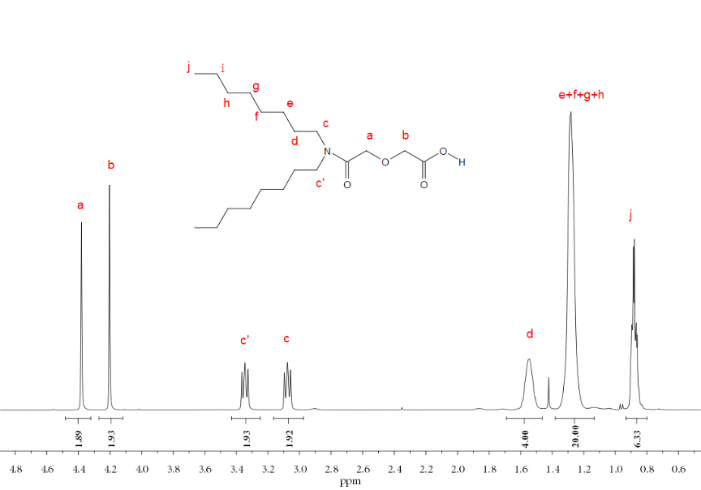


**Figure S2** ^1^H NMR spectrum of a1.

2.2 Synthesis of a2

A mixture of N,N’-dioctyldiglycolamic acid (35.7 g, 100 mmol), HOBT (14.9 g, 0.11 mol) and THF (100 mL) were mixed, a solution of THF with Boc-EDA (16 g, 0.1 mol) and DCC (22.7 g, 0.11 mol) was added slowly and he mixture was stirred over night at room temperature. After filtration, the solution was concentrated under reduced pressure and passed through an aluminum oxide column. Pure product was obtained as white solid (yield 61%).


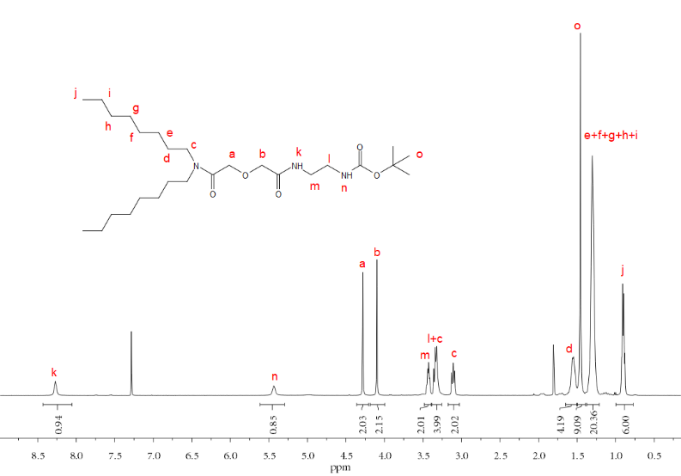


**Figure S3** ^1^H NMR spectrum of a2.

2.3 Synthesis of a3

Hydrochloric acid (10 mL) was added to the mixture of a2 (20 g, 0.05 mol) and ethyl acetate (60 mL). After 10h reaction at room temperature, the mixture was neutralized with saturated NaHCO3 solution. The organic phase was collected, dried with MgSO4, and evaporated under reduced pressure to give a crude product. The crude product was purified by passing through a silica gel column (yield 81%).


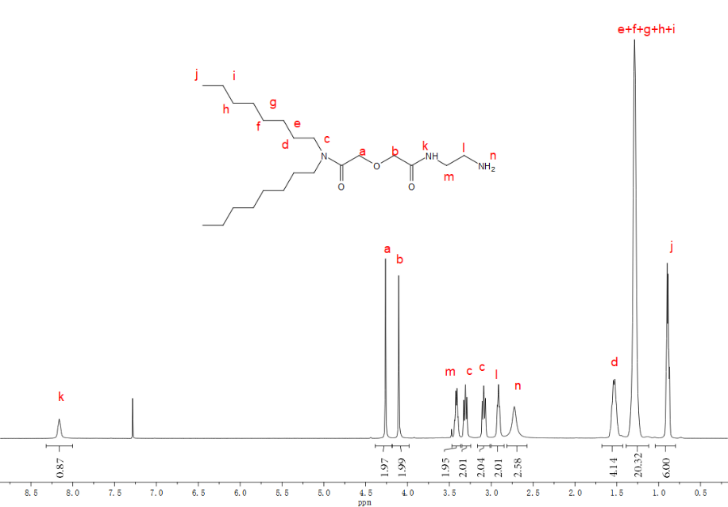


**Figure S4** ^1^H NMR spectrum of a3.

2.4 Synthesis M-a

To a solution of a3 (7.45 g, 18.0 mmol) and triethylamine (2.00 g, 19.8 mmol) in dichloromethane (120 mL) was added a solution of acryloyl chloride (2.61 g, 25.0 mmol) in dichloromethane (20 mL) at 0°C. The reaction mixture was brought to room temperature and stirred overnight. The solvent was washed successively with NaHCO_3_ solution and water. The organic layer was dried over anhydrous MgSO_4_ and concentrated under reduced pressure to give pure product (yield 49%).


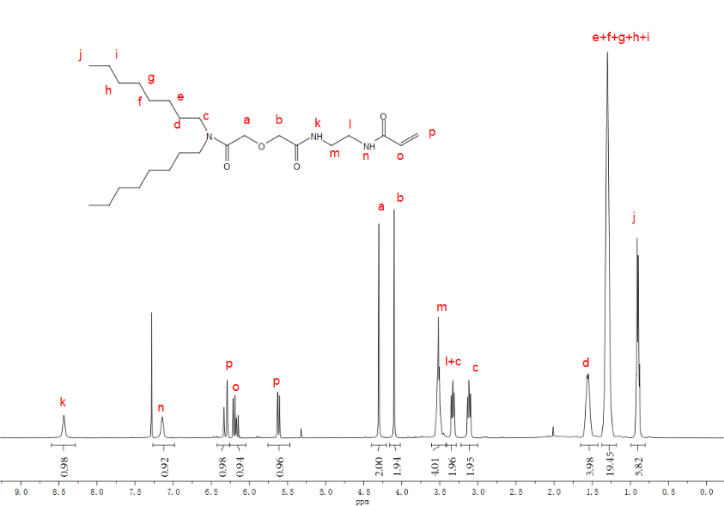


**Figure S5** ^1^H NMR spectrum of M-a.

2.5 Synthesis of M-b

M-b was synthesized as the method of M-a.


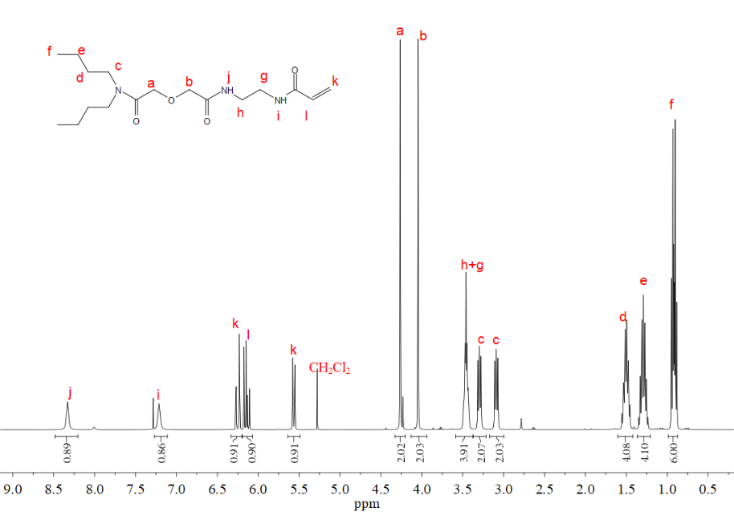


**Figure S6** ^1^H NMR spectrum of M-b.

2.6 Synthesis of M-c

M-c was synthesized as the method of M-a but with a little adjustment in that c1 was prepared in CH_2_Cl_2_ and c3 was extracted into the aqueous phase not the organic phase.


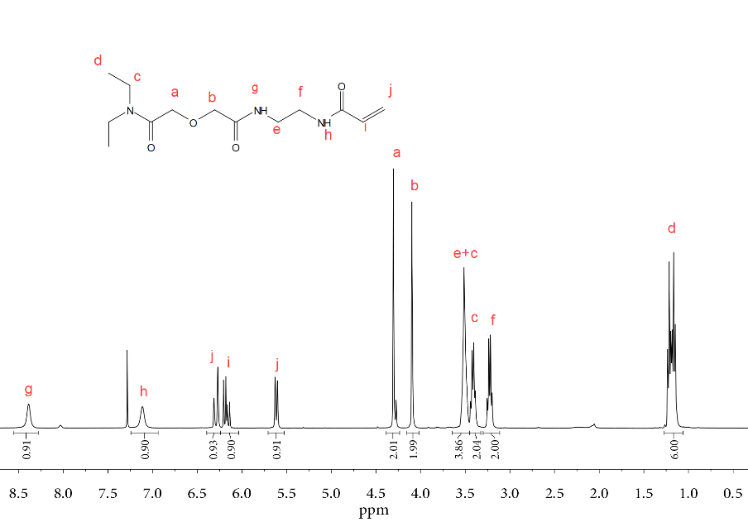


**Figure S7** ^1^H NMR spectrum of M-c.

**3 TG curves of S1, S2 and S3**





**Figure S8** TGA curves of APTES-SiO_2_(S1), ACVA-SiO_2_(S2) and polymer-grafted SiO_2_(S3).
